# Caffiene in Headache, (Migraine.)

**Published:** 1874-05

**Authors:** T. Curtis Smith

**Affiliations:** Middleport, Ohio


### CAFFIENE IN HEADACHE, (MIGRAINE.)

BY T. CURTIS SMITH, M.D., MIDDLEPORT, OHIO.

Migraine is the bane of happiness with very many persons. I will not stop here to discuss the opinions of its pathology, farther than to mention briefly some opinions held in regard to its nature. Ainstie holds it to be a strictly neurotic disease, generally occurring in persons who possess a conganital imperfection in the nerve centres especially the medulla oblongata, and that it is closely akin to or of the same class as epilepsy, asthma, angina pectoris, etc., and that it is only accidental whether the discrasia is manifested in one or the other of these forms of disease.*

*Practitioner for 1873.

Dr. D. N. Kinsman, of Ohio, believes it strictly a neurotic disease, having himself been a subject of it and a close observer of its manifestations.

Clifford Allbut denominates it a nervo-visceral disease.

Chambers and Dale consider it a disease, having its origin in derangements of the chylopoietic viscera.

For myself I believe it to he a strictly neurotic affection, having its primary origin in the nerve centres, especially the roots of the trigeminal nerve, but dissent from the view of Ainstie that it is strictly a neuralgia or that it has any connection direct or indirect with epilepsy, asthma, etc.*

My object in this paper is simply to show the value of caffiene in the treatment of this neurotic affection. Before going farther I will say that I deem nervous and sick headache as one and the same affection, modified in each case according to the temperament, age, habits and conditions of the various organs at the time of the attack. Premising this much, I will now state in brief the effects of caffiene on the physical economy.

It is justly classed among nervous stimulants of which, under certain conditions, it is one of our very best, while in other certain conditions it is very liable to do mischief. To explain, it is a cerebral stimulant and tends to increase the cerebral circulation, hence is of the most value as a nerve tonic, in those cases, where there is cerebral anemia and in those subjects in whom a determination of blood to the brain is not easily effected. It is therefore not a proper agent to administer where cerebral circulation is already too active. I have found it a most valuable nerve stimulant in many instances where formerly I had been in the habit of using such agents as valerian, tr. castor, musk, etc., and, by the way, much more reliable in its results, in well selected cases. An instance: Recently, I saw a patient in the night for a brother practitioner (Dr. N. D. Toby, of W. Columbia, W. Va.) a female, who had been greatly reduced by uterine hemorrhage. She was very feeble, exceedingly nervous and occasionally from accounts of her by the family, was on the point of swooning. A nerve stimulant seemed to be demanded (no hemorrhage now going on) and as she could not take any kind of

*For an expression of my views on this subject, see Medical and Surgical Reporter, of Philadelphia, Pa., for July 12, 1873, page 19.

alcoholic stimulant, I gave her caffiene, which had a happy effect, and on the Doctors arrival next morning the agent was continued. Had the condition of the patient been reversed, some other agent would have been required, as probably ammonia.

The following will, I think, determine the class of cases in which the agent was and was not found well adapted to its use, and are cases taken without selection from my practice and to whom it was administered before I became fully cognizant of its apparent influence over the cerebral determination.

The first case Mr. R——, aet 68, a hard laborer of steady habits, nervous temperament and good constitution for his age, has been all his life the subject of occasional attacks of sick headache (migraine for which he had tried many various remedies, but nothing seemed to answer in the way of relief, but the attacks were usually dissipated by a night's rest. I gave him caffiene at the outset of one of these attacks, which in a few minutes afforded marked relief. I thought little of it at the time, but when another attack occurred he requested another portion which again relieved him. Since that time he has kept it at hand and it has a great many times, either relieved or entirely prevented an attack. In fact he has not to my knowledge ever suffered much from headache since first using the agent, except once, when it could not be obtained. The attacks threaten as often as formerly, but do not culminate in the extreme pain, nausea, vomiting and great prostration that formerly attended or followed every recurrence. In fact, he does not now cease his labor for an attack but takes a dose of caffiene and works away as if in perfect health, nor does he loose a meal. In this instance the effects for good were remarkable and always the same.

Cases two and three were the daughters of the above gentleman, and doubtless inherited the disease. In both cases the agent affords great relief, but not always as well marked as in the case of the father. Very often however, the relief is prompt and effectual.

Case four.—Mrs. L., aet 34, of a highly nervous organization, spare build, habits of system regular, and health generally very good but suffers from migraine every few weeks. I had used for her on various occasions morphia sulp., which relieved the suffering but left ill sequential effects. I resorted to caffiene in July, 1873, for a severe attack from which she was suffering, during which great nausea came on which was not usual in her case. The effect of the agent, to use her own language was "charming." It seemed to greatly exhilerate the mind and left no bad effects behind it. In several subsequent attacks the caffiene was used with as much advantage as in the first instance.

Case five.—Mrs. C., aet 32, of nervo billious habit, spare build, system reduced in strength by several previous attacks of prolonged illness, married, two children, youngest aet 12 years, catamenia regular but painful, has anteverted uterus of many years standing, bowels always costive. This is one of the class of patients that will follow no plan of treatment more than a very brief period. She is the very frequent subject of migraine, caffiene has been used in her case four different times, but with the effect in every instance of apparently greatly increaseing the head pain and the nausea. She is not plethoric, nor of unusually active cerebral circulation, but the agent is useless in her case, the disease seeming to have its producing cause in the deranged uterine functions.* Morphia alone will afford partial relief, but is followed by vomiting, loss of appetite, etc., for a day or two.

Case six.—Mr. S., aet 36, of plethoric habit, hale and healthy in all respects, except an occasional attack of sick headache, during which he becomes a little pale, very languid and listless, usually vomits before relieved. I gave him caffiene with some misgiving as to the propriety of doing so, but noticing his more than usual palor he was ordered grain doses to be repeated every hour until better or decidedly worse. After the second dose he found himself quite relieved. Since this instance he has always found prompt and nearly perfect relief in a dose of grs. ii., and states that he had never before experienced any relief from any agent used, and many had been resorted to for that purpose.

Case seven.—Mrs. W., of West Virginia, aet 52, has for many years been a great sufferer from nervous headache. She is of nervous habit, broken in health, had used morphia with partial relief. She was ordered caffiene in grain doses every hour until relieved, usually one powder affords prompt and happy effects, and has so far never failed to do so.

*I believe a violation of natures laws regarding reproduction of the species, is the prime cause of trouble in this case.

Case eight.—Mr. B., aet 37, nervous habit, sedentary life, active brain worker, habits of system good, except not being perfectly temperate. Has frequent and extreme attacks of nervous migraine, of a most distressing character. I have ordered for him caffiene in several instances, but every time with the effect of greatly increasing the suffering. In this case nothing but morphia in very large portions will effect relief, and gives much the best results used hypodermically.

Case nine.—Mrs. M., aet 29, married, nervous habit, has four children, youngest, aet four years, a hard worker, habit of system good, with the single exception of occasional attacks of sich headache. She had resorted to so many agents for relief and without benefit that I had difficulty in persuading her to try the caffiene. The first portion, grs. ii., afforded very marked but not perfect relief, but by continuing to use the agent the force of the attacks seem to be well broken and a small dose now affords very good results, and attacks are far less frequent.

The number of these cases could be doubled or trebled in this report, but we deem it needless to give more.

The proportion of failures is about one in four in my hands, taking all the cases promiscuously, but by selection there is much better results. In those where it has failed, relief is usually found in some other agent, as morphia, or potass brom., or the two combined, nux vomica, quinia, or an emetic, according to the nature of the case.

It is scarcely necessary to say that in instances where the stomach is very foul or greatly acid, or any of the secretions greatly deranged, that this difficulty must be removed, but sometimes the caffiene will have a very marked effect for good, even while such difficulties are present.

I usually give from a half grain to two grains, according to the susceptibility of the patient to the action of remedies and according to the amount of tea and coffee habitually used, as those persons who use either of these agents to excess require a larger dose to effect them than those not using them or only doing so to a moderate extent.

I have not attempted to do more in this paper than simply give the results of the treatment of headache by the use of caffiene during the attack, thus leaving out of view most of the points in the physiological action of the agent, and many points in the general management of migrainous patients, which latter would alone afford material for a very extended article.

